# Supplementary material for: The Frequency and Function of NKG2C+CD57+ Adaptive NK Cells in Cytomagalovirus Co-Infected People Living with HIV Decline with Duration of Antiretroviral Therapy
Source: Viruses. 2023 Jan 24;15(2):323. doi: 10.3390/v15020323 (PMC9959045; doi:10.3390/v15020323)
Supplement: Supplementary file 1 [file viruses-15-00323-s001.zip › viruses-2172082-supplementary.pdf]

Supplemental Material

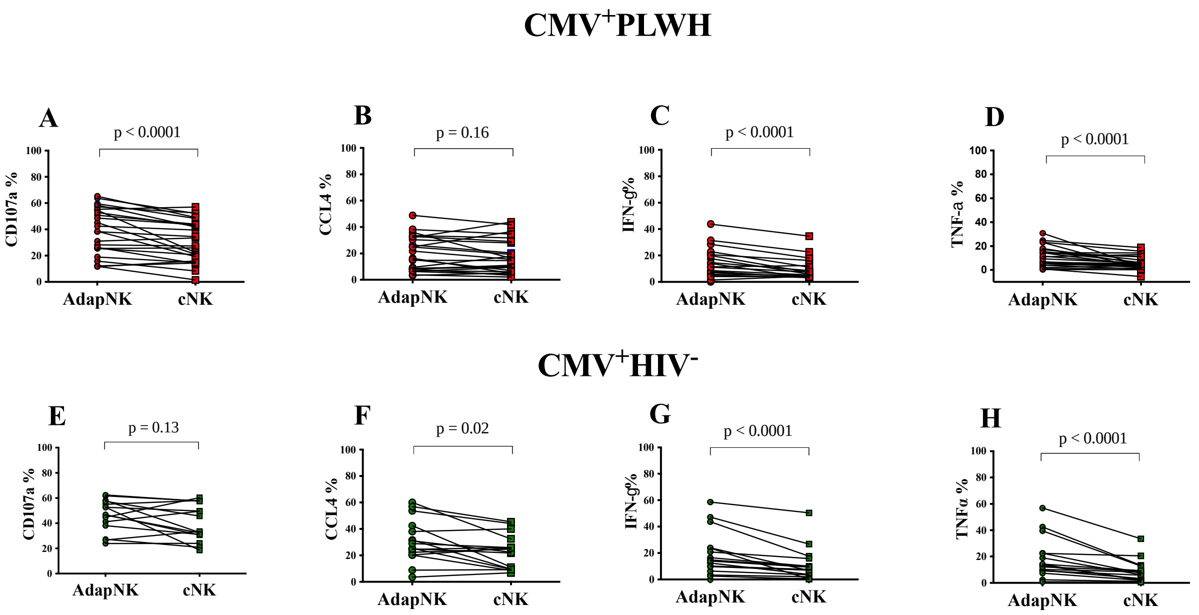

Figure S1. Differences in the frequency of functional adaptive versus conventional NK cells responding to stimulation with anti-HIV Envelope-specific antibody opsonized HIV-infected cells in CMV^+PLWH and CMV mono-infected (CMV^+HIV^-) subjects. (A-H) The y-axes show the frequency of adaptive NK cells and conventional NK cells from CMV^+PLWH (A-D) and CMV mono-infected (CMV^+HIV^-) persons (E-H) producing CD107a (A, E), CCL4 (B, F), IFN-γ (C, G) and TNF-α (D, H). Data points joined by a line show within- individual results. The significance of within-person differences in the frequency of functional adaptive NK cells versus conventional NK cells was assessed using Wilcoxon matched-pairs tests. *p*-values corresponding to these comparisons are shown over the lines linking the 2 data sets being compared.

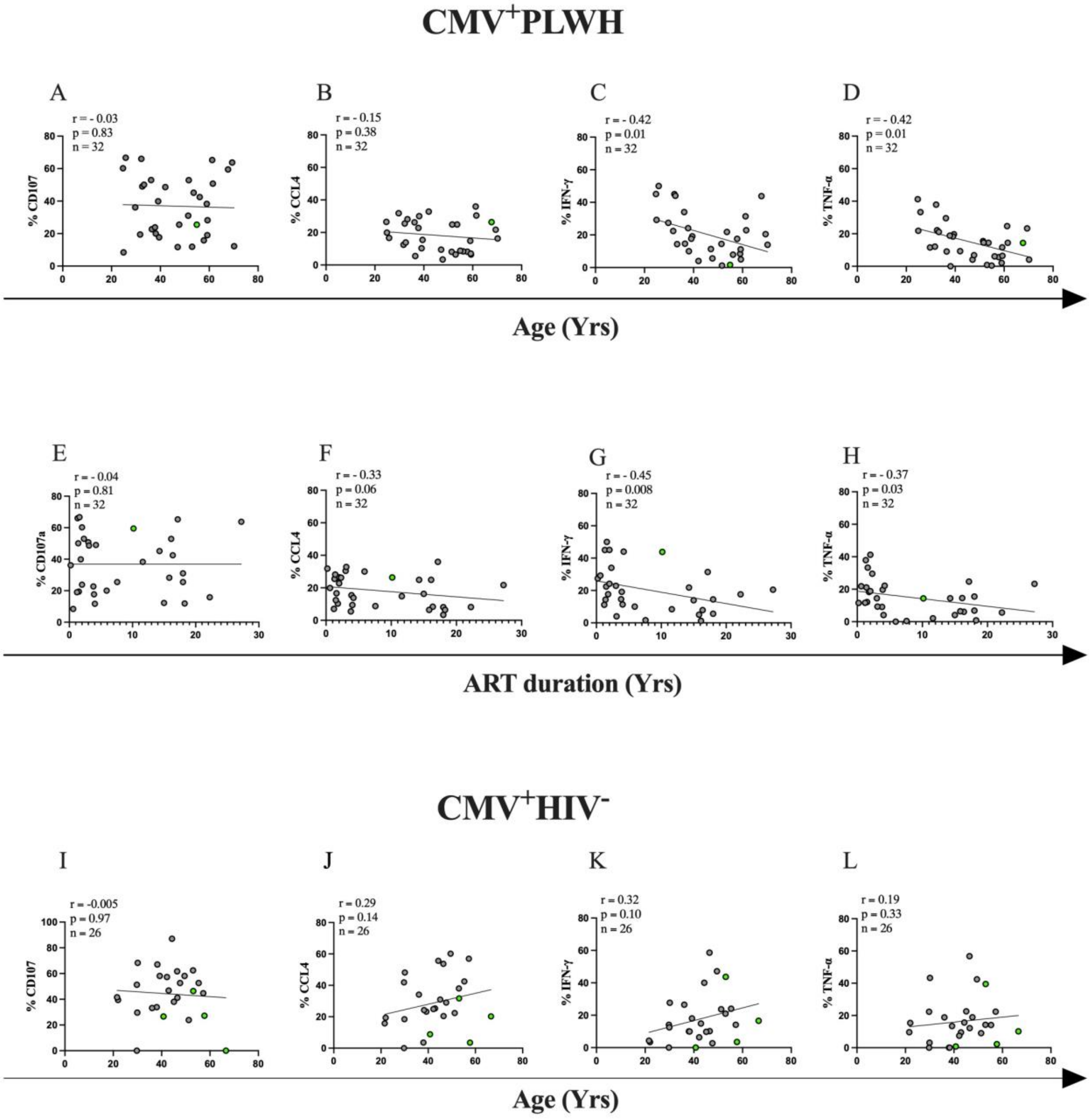

Figure S2. Correlations between the frequency of functional adaptive NK cells stimulated by antibody dependent NK cell activation with age and time on antiretroviral therapy (ART). (A-L) The y-axes show the frequency of functional adaptive NK cells responding to stimulation with anti-HIV Envelope-specific antibody opsonized HIV-infected cells. The x-axes show the ages (A-D, I-L) and duration of time on ART (E-H) for CMV^{+}PLWH (A-H) and CMV mono-infected (CMV^{+}HIV^{-}) (I-L) participants. The responsiveness of adaptive NK cells to antibody dependent stimulation was assessed by measuring the frequency of these cells externalizing CD107a (A, E,

I) or secreting CCL4 (B, F, J), IFN-γ (C, G, K) or TNF-α (D, H, L). The significance of correlations between the frequency of stimulated functional adaptive NK cells and age/ART duration was assessed using Spearman correlation tests. The correlation coefficients (r), the *p*-values, and the number of subjects tested for each correlation are shown in the top left corner of the graphs. Data points corresponding to results attributed to females are distinguished from those attributed to males by being shown in light green.
